# Interference of Illusory Contour Perception by a Distractor

**DOI:** 10.3389/fpsyg.2021.526972

**Published:** 2021-06-11

**Authors:** Junkai Yang, Lisen Sui, Hongyuan Wu, Qian Wu, Xiaolin Mei, Xiang Wu

**Affiliations:** ^1^Laboratory for Behavioral and Regional Finance, Guangdong University of Finance, Guangzhou, China; ^2^Department of Psychology, Sun Yat-sen University, Guangzhou, China; ^3^Department of Neurosurgery, Guangdong Provincial Hospital of Chinese Medicine, The Second Affiliated Hospital of Guangzhou University of Chinese Medicine, Guangzhou, China

**Keywords:** illusory contour, distractor, task difficulty, perception, attention

## Abstract

The visual system is capable of recognizing objects when object information is widely separated in space, as revealed by the Kanizsa-type illusory contours (ICs). Attentional involvement in perception of ICs is an important topic, and the present study examined whether and how the processing of ICs is interfered with by a distractor. Discrimination between thin and short deformations of an illusory circle was investigated in the absence or presence of a central dynamic patch, with difficulty of discrimination varied in three levels (easy, medium, and hard). Reaction time (RT) was significantly shorter in the absence compared to the presence of the distractor in the easy and medium conditions. Correct rate (CR) was significantly higher in the absence compared to the presence of the distractor in the easy condition, and the magnitude of the difference between CRs of distracted and non-distracted responses significantly reduced as task difficulty increased. These results suggested that perception of ICs is more likely to be vulnerable to distraction when more attentional resources remain available. The present finding supports that attention is engaged in perception of ICs and that distraction of IC processing is associated with perceptual load.

## Introduction

**FIGURE 1:**
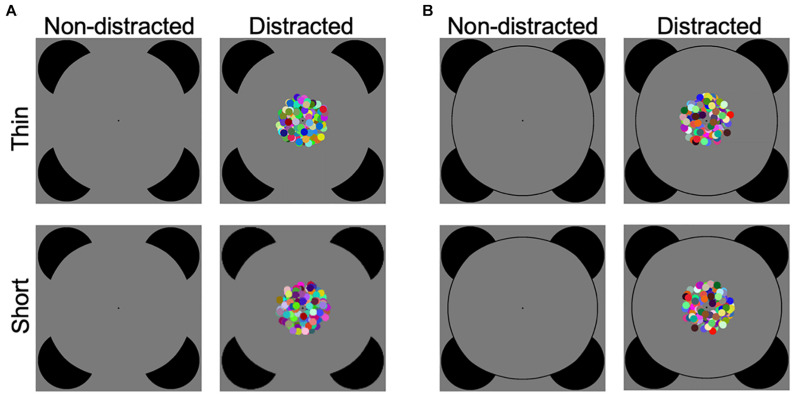
Illustration of experimental stimuli. **(A)** The illusory contour (IC) discrimination task. Participants were required to discriminate between thin and short deformations of a standard Kanizsa-type circle. There were three levels (easy, medium, and hard) of task difficulty. Task difficulty was manipulated by varying the extent of deformation, and large deformation is shown for illustration purpose (see Supplementary Figure 1 for illustration of illusory contour stimuli in all difficulty conditions). The target stimuli were presented in the absence (non-distracted) or presence (distracted) of a central dynamic patch. **(B)** The control real contour (RC) discrimination task, which was the same as the illusory contour discrimination task except that the circle was drawn with the physical line.

One of the essential functions of the visual system is to recognize objects even when object information is widely separated in space, as revealed by the well-known Kanizsa-type illusory contours (ICs) in which a contour is perceived despite the fragments of the contour being separated by gaps (Kanizsa, 1976; Figure 1A).

Because the Kanizsa-type IC appears to be perceived effortlessly, it has been considered to arise from pre-attentive or automatic processes. In a seminal work by Davis and Driver (1994) using a visual search task, an IC figure among non-IC figures was found to be detected in parallel without requirement of attention. The parallel search of ICs has been found in later studies (Davis and Driver, 1998; Herrmann and Mecklinger, 2000; Senkowski et al., 2005; Takahashi et al., 2007), and is in line with the findings from neuropsychological studies of neglect patients that showed preserved IC perception when parts of IC figures were presented in the neglected hemisphere (Mattingley et al., 1997; Vuilleumier et al., 2001; Conci et al., 2009). Besides the classic visual search task, other paradigms were also adopted in research of attentional involvement in IC perception. For example, using a multiple object tracking task, it was found that the task performance was impaired when ICs were formed by targets and distractors, and was improved when ICs were formed by targets (Keane et al., 2011). This suggests that the IC is formed and directs attention automatically.

There were also visual search studies reporting that reaction times (RTs) searching for ICs increased with display size when the non-IC distractors were similar to the IC target figure (Gurnsey et al., 1996; Conci et al., 2007, 2011; Wiegand et al., 2015; Nie et al., 2016), or when distractors were also IC figures but with shapes different from the shape of the target (Li et al., 2008). Moreover, while a single figure was required to be perceived in previous IC studies of neglect patients (Mattingley et al., 1997; Vuilleumier et al., 2001; Conci et al., 2009), a recent neglect study employed a visual search task and showed that attentional competition between multiple objects (i.e., target and distractor figures) limited perceiving ICs in the neglected hemisphere, indicating a contribution of attention to IC processing (Gögler et al., 2016). In addition, by using a task that required detection of removal of quarter-segments from circles, a study of extinction patients showed that, while extinction was absent when grouping in IC shapes extended from the attended hemifield, extinction was not diminished when the grouping propagated from the unattended hemifield (Conci et al., 2018). This suggests that attention is crucial for IC processing. These results indicating attentional involvement in IC processing are in agreement with a view that high-level cognitive processes are necessary for perception of ICs (Coren, 1972; Gregory, 1972; Rock and Anson, 1979; Wu et al., 2015).

Previous studies have mainly focused on investigating attraction of attention or modulation of attention allocation by ICs (e.g., Davis and Driver, 1994; Vuilleumier et al., 2001; Senkowski et al., 2005; Conci et al., 2007; Keane et al., 2011). Direct examination of interference of IC perception by a distractor is almost non-existent (although see Rauschenberger and Yantis, 2001). Distraction studies focus on whether a process is vulnerable or immune to a distractor and provide another way to assess attentional requirement of a process (Lavie, 1995; Forster and Lavie, 2008; Lavie and Dalton, 2014). For example, an automatic process would be less influenced by a distractor and a process that requires more attentional resources would be more vulnerable to distraction (Lavie and Dalton, 2014). In the distraction research, a perceptual load theory suggests that processing of distractors is determined by perceptual load of relevant information, and supporting findings show that distractors’ interference is observed in the lower load conditions in which more attentional resources remain available for relevant targets (Lavie, 1995).

Illusory contour figures are formed by inward inducers, and control figures are typically formed by outward inducers. It has been debated whether the difference between the IC and control figures reflects the grouping of inducers or the non-grouping processes that are related to the local processing of inducers (for review, see Seghier and Vuilleumier, 2006; Anderson, 2007). This would also be an issue when addressing attentional involvement of IC perception. For example, in a classic visual search task, distractors are not irrelevant to the target given that the target may potentially appear in distractors’ locations. When an IC figure is searched among outward-inducer control figures, it would be difficult to determine whether the results indeed indicate involvement of attention in the grouping process (Gurnsey et al., 1996). To address the controversy about whether IC shape discrimination tasks reflect the completion process (Seghier and Vuilleumier, 2006; Anderson, 2007), a recent study by Yang et al. (2015) adopted a thin/short task (Pillow and Rubin, 2002) and devised a new paradigm using a circle shape instead of the commonly used square or triangle shape. The results of Yang et al. (2015) proved that it was hard for participants to perform the Kanizsa discrimination task by relying on the rotation information of inducers, suggesting that the shape discrimination task indeed taps into the effect of completion processing. Moreover, for attention research, distractors have been designed to be less task-relevant to the target (Forster and Lavie, 2008), which would be helpful in investigation of attentional involvement in IC perception.

The present study thus aimed to examine interference of IC perception by a distractor, with varied perceptual load. (1) The completion process in IC perception is investigated using the thin/short IC shape discrimination task. The inducers of a standard Kanizsa circle were rotated to produce a thin deformation and a short deformation, and the participants were required to discriminate between the two types of deformations (Ringach and Shapley, 1996; Pillow and Rubin, 2002; Yang et al., 2015; Figure 1A). Ringach and Shapley (1996) devised a method for measuring the compelling perceptual completion in IC perception. This thin/fat shape discrimination task (Ringach and Shapley, 1996) requires classifying deformations of a standard Kanizsa-type square as thin or fat shapes (also see Victor and Conte, 2000; Spehar and Clifford, 2003 for another objective measure of IC formation by discriminating between portrait and landscape shapes). Pillow and Rubin (2002) introduced a thin/short task when examining perceptual completion across the vertical meridian, which is a variation of the original thin/fat task and allows separate investigations of the vertical and horizontal IC boundaries. Yang et al. (2015) adopted the thin/short paradigm and used a standard circle instead of the standard square, which was adopted in the present study (see Figure 1 in Yang et al., 2015 for an illustration of these variations of the IC shape discrimination task). (2) The interference of a distractor on the IC task was examined. The Kanizsa figure, i.e., the target stimulus, was displayed in the absence or presence of a centrally presented dynamic patch, i.e., the distractor stimulus. The participants were asked to perform the discrimination task while ignoring the dynamic patch. The dynamic patch was irrelevant to the discrimination task in two aspects. First, unlike the distractors consisting of outward inducers, the dynamic patch would bear no perceptual relevance to the target IC figure. Second, the central dynamic patch and the peripheral Kanizsa-type circle were widely separated in space and thus would be spatially irrelevant. To avoid a potential issue that lack of interference is because a distractor is not salient enough, the present dynamic patch was constructed with colored dots moving in random directions. This was based on the findings that colored and moving objects are more salient than stationary gray stimuli (Abrams and Christ, 2003; Tsuchiya and Koch, 2005; Chua, 2014; von Mühlenen and Conci, 2016). (3) Perceptual load of the IC task was varied. Difficulty of the IC discrimination task was manipulated in three levels (easy, medium, and hard) by varying the extent of deformation (Ringach and Shapley, 1996; Pillow and Rubin, 2002; Yang et al., 2015). This would represent the modulation of perceptual load, with the easy condition corresponding to the low load condition (Lavie, 1995). Together, the current study investigated interference of a distractor (the factor distraction, without or with the distractor) on an IC perceptual task, while perceptual load of the IC task varied (the factor task difficulty, in easy, medium, and hard levels). We predicted that the IC discrimination performance would be interfered with by the distractor and that the interference effect would be more likely to be observed in the easier conditions, as suggested by the perceptual load theory (Lavie, 1995). In other words, we hypothesized that IC processing would be more likely to be vulnerable to distraction when more attentional resources remained available in the lower load conditions.

Moreover, there is a long research history about whether perception of Kanizsa-type ICs and real contours (RCs) involves common mechanisms (Hirsch et al., 1995; Larsson et al., 1999; Mendola et al., 1999; Lee, 2001; Ramsden et al., 2001; Maertens and Pollmann, 2005; Li et al., 2008; Kinsey et al., 2009; Pan et al., 2012). Therefore, in a control task, we also investigated discrimination between thin and short deformations of a corresponding real circle using the current distraction paradigm (Figure 1B).

## Materials and Methods

### Participants

Thirty participants (eight males, mean age ± *SD* 21.7 ± 2.9 years) participated in the IC discrimination task and the control RC discrimination task. A screening procedure was conducted before the formal experiment to test whether a participant could perform the discrimination task (see below). Eight additional participants showed great difficulty in completing the tasks and thus did not perform the formal experiment (Pillow and Rubin, 2002; Yang et al., 2015). All participants were naive to the experiments. All participants had normal or corrected-to-normal vision. The research protocols in this study followed the tenets of the Declaration of Helsinki and were approved by the Institutional Review Board of the Psychology Department at Sun Yat-sen University. All participants gave written informed consent.

### Power Analysis

The key question asked in the present study was whether IC perception could be interfered with by a task-irrelevant distractor, as indicated by the difference between performances of distracted and non-distracted responses. RTs were used in the power analysis given that RTs have been suggested to be more sensitive than correct rates (CRs) in investigating the interference effect (Beck and Lavie, 2005; Bahrami et al., 2007). *A priori* power analysis was performed using G^∗^Power 3 (Faul et al., 2007) to examine the required sample size to detect the interference effect. For investigation of the interference effect, because the present Kanizsa-type circle figures were adopted from a recent work (Yang et al., 2015) and a novel dynamic patch was used as the distractor, the size of the interference effect was in accordance with the effect observed in a pilot experiment (mean RT difference = 43.27 ms; Cohen’s *d_z_* = 0.97; see section “Pilot Experiment 3” in the Supplementary Material for details). Given this effect size, the alpha level of *p* < 0.05 (two tailed), and the power of 0.8, the required sample size was 11. We also performed *a priori* power analysis for the two-way interaction between the interference effect (without or with the distractor) and task difficulty (easy, medium, and hard) (see detailed descriptions below). Due to the lack of previous data on the interaction, the size of the interaction was determined *a priori* assuming a moderate effect size *f* = 0.25 (Cohen, 2013). Given this effect size, the alpha level of *p* < 0.05 (two tailed), and the power of 0.8, the required sample size was 19. Therefore, the current sample size would have sufficient power for investigation of the interference effect and the interaction between the interference effect and task difficulty.

### Stimuli and Procedure

The experiments proceeded in a quiet and dim room where the participants sat in front of a Gimit i5 4590/GTX750Ti desktop computer equipped with an AOC G2460PQU/BR LCD computer monitor (120 Hz refresh rate, 1920 × 1080 resolution, and 53.1 cm × 29.8 cm) and a Logitech M90 computer mouse. The viewing distance was 50 cm, and a chinrest was used to stabilize the head. Luminance of the stimulus was measured by a TES-137 Luminance Meter.

**FIGURE 2:**
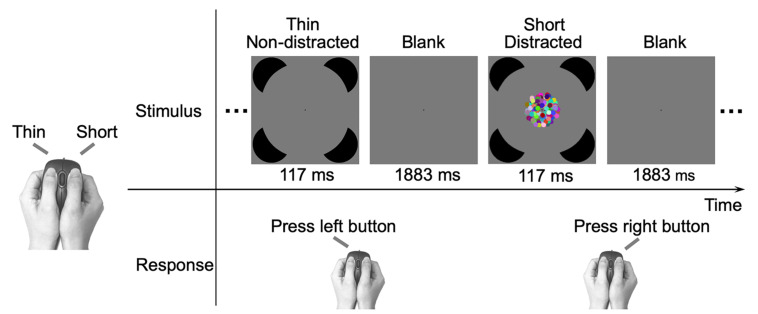
Illustration of the experimental procedure. Each trial consisted of a 117 ms figure and a 1883 ms blank. The participants were required to respond to the figure with a left or right thumb button-press as quickly and accurately as possible. In the shown example, two successive trials in the IC task (Figure 1A) are presented and participants made a “thin” response using the left thumb or made a “short” response using the right thumb. The relation between the stimulus type and responding hand is depicted on the left, and the assignment of hands was counterbalanced across participants. In the control RC task (Figure 1B), participants made the same “thin” or “short” response as in the IC task.

The study included an IC task and a control RC task. In the IC task, the participants were instructed to discriminate between thin and short deformed Kanizsa circle figures as quickly and accurately as possible, by pressing one of the two buttons of the computer mouse using their thumbs (the mouse served as a responding box held by the two hands) (Figure 2). The assignment of corresponding hands was counterbalanced across participants. The deformed figures were constructed from a standard Kanizsa circle figure by a procedure that has been described in detail in Yang et al. (2015). In short, the deformation procedure rotated inducer mouth edges of the standard figure (Ringach and Shapley, 1996; Pillow and Rubin, 2002), with an additional smoothing step that was applied to further reduce the non-smoothness of the inducer mouth (see Supplementary Figure 2 in Yang et al., 2015 for the detailed illustration of the deformation procedure). Thus, the contour fragments of Kanizsa circle figures provided fewer cues for the discrimination. The support ratio (i.e., the ratio of the edge length supported by the inducers to the total edge length of the contour) was 0.5. The diameter of the inducer and the standard circle subtended visual angles of 9.8° and 25.4°, respectively. The left/right (vertical) and top/bottom (horizontal) contours were varied (Pillow and Rubin, 2002) to produce the thin and short deformations, respectively. The extent of deformation was indicated by the rotation angle, which was defined as the deviation angle between the inducer mouth edges of the standard and deformed figures. Three rotation angles of 4.8°, 3.0°, and 2.0° were used, representing the easy, medium, and hard discrimination conditions, respectively. The inducers were of a black color (RGB values: 0 0 0; luminance: 0.31 cd/m^2^). The stimuli were displayed on a gray (RGB values: 127 127 127; luminance: 18.09 cd/m^2^) background with a black fixation point (radius of 0.2°) permanently displayed at the center of the screen, and the participants were asked to fixate on the fixation point.

The Kanizsa figures were presented for 117 ms in a random order and were interleaved with a blank screen of 1883 ms. For half of the Kanizsa figures, a dynamic patch was simultaneously presented at the center for 117 ms serving as the distractor. The dynamic patch consisted of 140 colored dots with the same radius of 0.6°. For each of the red, green, and blue components, the values of the 140 dots were generated from a uniform distribution of values 0–255. The mean luminance of the dynamic patch was 35.00 cd/m^2^. In the presentation, the dots’ initial positions were randomly assigned (so that the edge of the patch was irregular and provided no clues for the target shape discrimination task) within a virtual annulus between radiuses of 4.8° and 0.6°. Each dot rotated around the fixation point either clockwise or counterclockwise, with an angular speed of 3.6°/second (i.e., moved 0.4° in the 117 ms presentation).

There were three blocks. Each block contained 96 stimuli, in which the six types of stimuli (non-distracted or distracted stimuli in three difficulty levels) were randomly mixed and each stimulus type was presented 16 times (48 times in total in three blocks).

The control RC task was the same as the IC task except for the following differences. (1) The RC figures were produced from the IC figures by drawing contours between inducers using additional black lines (thickness of five pixels). The shape of the physical contour was constructed to be a circular arc tangent to the edges of the inducer (Ringach and Shapley, 1996), which followed the proposal that IC shapes are approximated by smoothly joined circular arcs (Ullman, 1976). (2) The rotation angles of 3.6°, 2.0°, and 1.2° were used for the RC figures (which were smaller than those for the IC figures), with an attempt to reduce differences in discrimination accuracy between the two contour types.

A group of 30 participants performed the IC task and the control RC task, which were arranged in two separate sessions, and their order was counterbalanced across participants. Before the formal task, the stimuli and the task were explained to the participants with a written instruction. Moreover, a screen procedure was conducted before the formal experiment. For each task, the screen procedure was the same as the formal task except that there were two (instead of three) blocks and each block contained 48 (instead of 96) stimuli. Eight additional participants reported great difficulty with performing the task (their discrimination accuracy was lower than 75% in the easy non-distracted condition in one of the two tasks), and therefore did not further perform the formal experiment (Pillow and Rubin, 2002; Yang et al., 2015).

In addition, three pilot experiments were conducted before the formal experiments to test the validity of the current experimental design as well as the appropriateness of corresponding experimental parameters (see details in section “Pilot Experiments’ in the Supplementary Material).

The stimuli were presented using Psychtoolbox 3.0.12 for Matlab^1^ running on Windows 7^2^. The setup and programming of Psychtoolbox in the present study followed the procedures as recommended on the Psychtoolbox website to achieve precise timing of stimulus presentation (e.g., using double buffer for visual presentation). For more details, see Kleiner et al. (2007) and text footnote 1. Moreover, the program was set to run under the highest priority to thereby avoid interruptions by other background processes. MATLAB R2010b (The Mathworks, Natick, MA, United States) was used to present stimuli, and collect and analyze the data.

### Data Analyses

Reaction time and discrimination accuracy (CR) were measured. Missing responses and responses with RTs longer than 1000 ms were excluded from analysis for each participant. On average, 3.2 ± 2.0 and 3.0 ± 2.2 responses were excluded from the IC task and the control RC task, respectively. The RT analysis included only correct responses. The results of log(RT) are presented in Supplementary Figure 2, showing consistent results. The RTs and CRs of all conditions (distraction: without or with the distractor; task difficulty: easy, medium, and hard) in both tasks were checked for normality using the Kolmogorov–Smirnov (K–S) test, with K–S *p* > 0.05 indicating that a variable was not significantly different from the normal distribution. For all conditions in both tasks, the RTs and CRs satisfied the normality assumption (K–S *p* > 0.05). Greenhouse–Geisser corrections were applied to the repeated measures analysis of variance (ANOVA) with the factor distraction (without or with the distractor) and the factor task difficulty (easy, medium, and hard). Bonferroni corrections were applied to all *t*-tests, and corrected *p*-values ≤ 0.05 were considered significant. All *t*-tests were two-tailed.

## Results

The study included an IC discrimination task and a control RC discrimination task. In the IC task, discrimination between the thin and short deformations of a standard Kanizsa-type circle was performed in the absence (non-distracted) or presence (distracted) of a central dynamic patch. Discrimination difficulty was modulated in three levels (easy, medium, and hard) by varying the extent of deformation (discrimination was easier for larger deformation, and vice versa). The control RC task was similar to the IC discrimination task except that real instead of ICs were used. RTs and CRs of the responses were measured. The difference between the RTs or the CRs of distracted and non-distracted responses was referred to as the RT cost or the CR cost, indicating the interference effect.

### Illusory Contour Task

**FIGURE 3:**
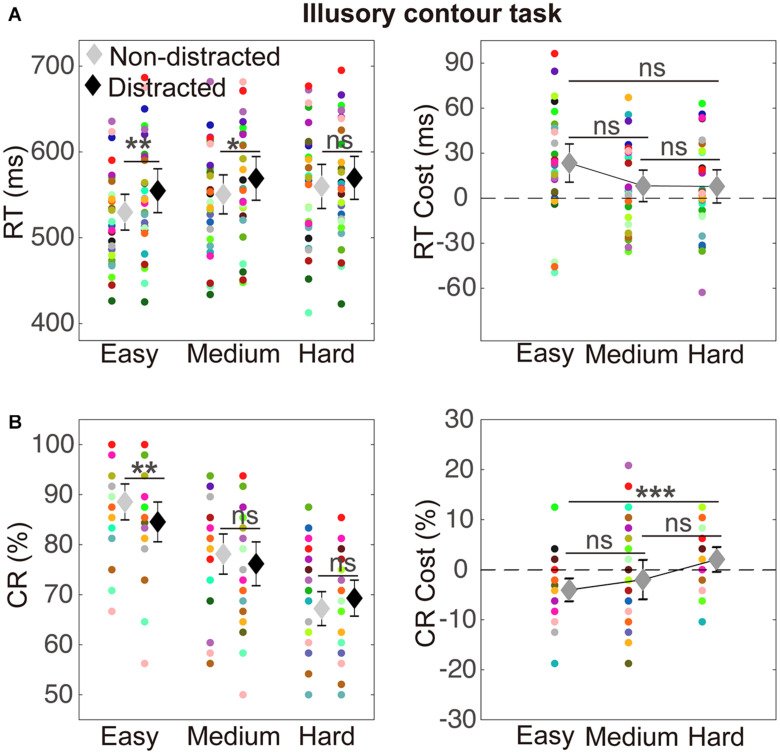
Illustration of results in the illusory contour (IC) task. **(A)** The results of reaction times (RT). RTs of non-distracted and distracted responses at the three difficulty levels (easy, medium, and hard) are shown on the left. RT costs (distracted minus non-distracted) that indicated interference effects are shown on the right. Significance of paired *t* tests with Bonferroni correction was indicated: ns, *p* > 0.05; *p < 0.05; **p < 0.01; and ***p < 0.001. The mean RTs are indicated by the diamond markers, with error bars indicating 95% confidence intervals. The data from individual subjects are indicated by the circle markers in different colors, which indicate different subjects. **(B)** The results of correct rates. CR cost refers to the difference between CRs of distracted and non-distracted responses. Other conventions are as in **(A)**.

The RT results are depicted in Figure 3A. A two-way ANOVA with within-subject factors of distraction (without or with the distractor) and task difficulty (easy, medium, and hard) was performed. The ANOVA showed a significant main effect of distraction [*F*(1,29) = 15.15, *p* = 0.001, η_p_^2^ = 0.34] and a significant main effect of task difficulty [*F*(2,58) = 19.44, *p* < 0.001, η_p_^2^ = 0.40]. The interaction between the two factors was not significant [*F*(2,58) = 1.94, *p* = 0.154, η_p_^2^ = 0.06]. A significant RT cost was observed in the easy condition (mean difference = 25.02, *t_29_* = 3.75, *p*_corrected_ = *0.003*) and the medium condition (mean difference = 18.63, *t_29_* = 2.99, *p*_corrected_ = *0.018*). Detailed values of RTs of distracted and non-distracted responses and comparisons between them are listed in Table 1.

**TABLE 1:** RTs and CRs of distracted and non-distracted responses and comparisons between them in the IC task.

	**Non-distracted**		**Distracted**		**Comparisons (distracted versus non-distracted)**				
**Difficulty**	**Mean**	**95% CI**	**Mean**	**95% CI**	**Mean difference**	***95%* CI**	***t***	***p* corrected**	**Cohen’s *d*_z_**
**RT (ms)**									
Easy	529.76	(508.89, 550.62)	554.77	(529.16, 580.39)	25.02	(11.37, 38.66)	3.75	0.003	0.68
Medium	550.52	(527.80, 573.23)	569.15	(543.63, 594.66)	18.63	(5.89, 31.37)	2.99	0.018	0.55
Hard	559.73	(533.98, 585.49)	569.77	(544.74, 594.81)	10.04	(−2.61, 22.70)	1.62	0.345	0.30
**CR (%)**									
Easy	88.54	(84.95, 92.14)	84.55	(80.57, 88.53)	−3.99	(−6.28, −1.71)	3.76	0.003	0.65
Medium	78.13	(74.11, 82.14)	76.18	(71.81, 80.55)	−1.94	(−5.88, 1.99)	1.01	> 0.250	0.18
Hard	67.22	(63.83, 70.61)	69.31	(65.70, 72.91)	2.08	(−0.39, 4.56)	1.72	> 0.250	0.31

*Conventions are as in Figure 3.*

The CR results are depicted in Figure 3B. A two-way ANOVA with within-subject factors of distraction (without or with the distractor) and task difficulty (easy, medium, and hard) showed a significant main effect of task difficulty [*F*(2,58) = 122.92, *p* < 0.001, η_p_^2^ = 0.81] and a significant interaction between the two factors [*F*(2,58) = 5.32, *p* = 0.017, η_p_^2^ = 0.16]. The main effect of distraction was not significant [*F*(1,29) = 1.75, *p* = 0.196, η_p_^2^ = 0.06]. A significant CR cost was observed in the easy condition (mean difference = −3.99, *t_29_* = 3.76, *p*_corrected_ = 0.003). Detailed values of CRs of distracted and non-distracted responses and comparisons between them are listed in Table 1.

### Control Real Contour Task

**FIGURE 4:**
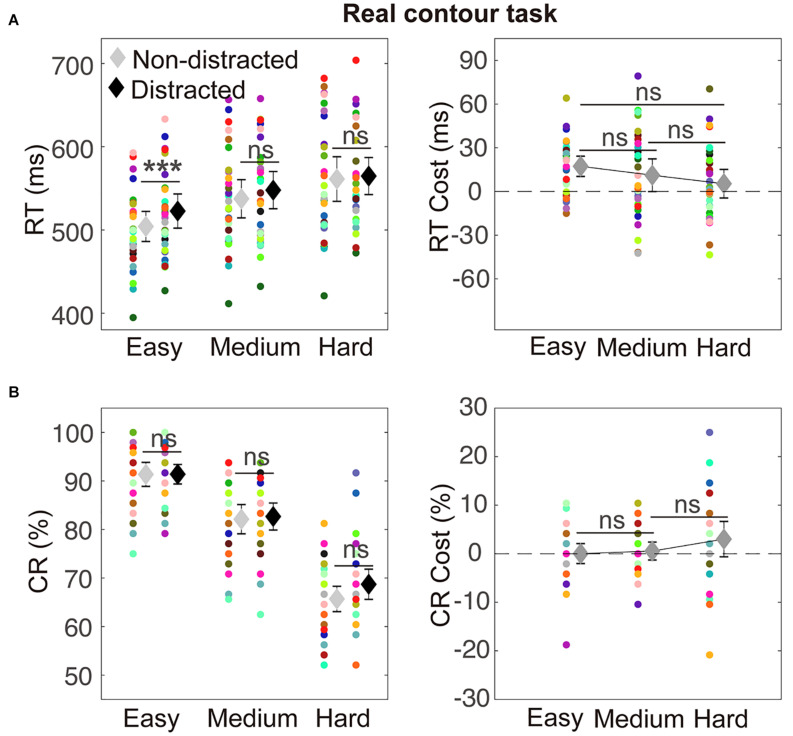
Illustration of results in the control real contour (RC) task. Conventions are as in Figure 3.

The RT results are depicted in Figure 4A. A two-way ANOVA with within-subject factors of distraction (without or with the distractor) and task difficulty (easy, medium, and hard) was performed. The ANOVA showed a significant main effect of distraction [*F*(1,29) = 8.02, *p* = 0.008, η_p_^2^ = 0.22] and a significant main effect of task difficulty [*F*(2,58) = 67.08, *p* < 0.001, η_p_^2^ = 0.70]. The interaction between the two factors was not significant [*F*(2,58) = 3.04, *p* = 0.057, η_p_^2^ = 0.10]. A significant RT cost was observed in the easy condition (mean difference = 18.47, *t*_29_ = 5.02, *p*_corrected_ < 0.001). Detailed values of RTs of distracted and non-distracted responses and comparisons between them are listed in Table 2.

**TABLE 2:** RTs and CRs of distracted and non-distracted responses and comparisons between them in the control RC task.

	**Non-distracted**		**Distracted**		**Comparisons (distracted versus non-distracted)**				
**Difficulty**	**Mean**	**95% CI**	**Mean**	**95% CI**	**Mean difference**	***95%* CI**	***t***	***p* corrected**	**Cohen’s *d*_*z*_**
**RT (ms)**									
Easy	504.21	(486.13, 522.29)	522.68	(502.14, 543.21)	18.47	(10.94, 26.00)	5.02	<0.001	0.92
Medium	537.44	(514.45, 560.43)	547.81	(525.50, 570.12)	10.37	(−1.08, 21.81)	1.85	0.222	0.34
Hard	561.14	(534.39, 587.90)	564.76	(542.49, 587.03)	3.61	(−8.53, 15.76)	0.06	>0.250	0.11
**CR (%)**									
Easy	91.35	(88.87, 93.83)	91.39	(89.38, 93.40)	0.04	(−2.02, 2.09)	0.03	> 0.250	0.01
Medium	82.12	(79.11, 85.12)	82.67	(79.89, 85.45)	0.56	(−1.29, 2.40)	0.62	>0.250	0.11
Hard	65.69	(63.08, 68.31)	68.72	(65.61, 71.82)	3.02	(−0.63, 6.67)	1.69	>0.250	0.31

*Conventions are as in Figure 4.*

For the CRs, a two-way ANOVA with within-subject factors of distraction (without or with the distractor) and task difficulty (easy, medium, and hard) showed a significant main effect of task difficulty [*F*(2,58) = 190.89, *p* < 0.001, η_p_^2^ = 0.87)]. The main effect of factor distraction [*F*(1,29) = 2.25, *p* = 0.145, η_p_^2^ = 0.07] and the interaction between the two factors [*F*(2,58) = 1.65, *p* = 0.204, η_p_^2^ = 0.05] were not significant. None of the three difficulty levels showed a significant CR cost (detailed values of CRs of distracted and non-distracted responses and comparisons between them are listed in Table 2).

### Direct Comparison Between the IC and Control RC Tasks

In the results presented above, a significant RT cost was observed in the easy condition in the control RC task, similarly to that in the IC task. The CR cost was not significant in all the three task difficulty levels in the control RC task, whereas in the IC task, a significant CR cost was observed in the easy condition and the magnitude of the CR cost significantly reduced as task difficulty increased.

To further explore the potential similarity and discrepancy in the results between the IC and control RC tasks, a three-way ANOVA analysis across the IC and control RC tasks was carried out (see below). An assumption was that a significant three-way interaction could indicate a difference in the modulation of distraction by task difficulty between the two tasks.

Here, we should emphasize that such analyses directly comparing the IC and control RC tasks were exploratory analyses. The main theoretical motivation and conclusion of the present study were in regard of the IC task. The RC task was carried out as a control task with respect to a further interest of possible common IC and RC mechanisms, and the current study was not designed with sufficient power to directly compare the two tasks. Therefore, the direct comparison analysis between the IC and control RC tasks was exploratory, and the corresponding results and interpretations would be considered as suggestive but not conclusive.

A three-way ANOVA with factors distraction (without or with the distractor), task difficulty (easy, medium, and hard), and task type (IC and control RC tasks) was performed. For the RT data, the results showed a significant main effect of distraction [*F*(1,29) = 17.50, *p* < 0.001, η_p_^2^ = 0.38], a significant main effect of task difficulty [*F*(2,58) = 60.56, *p* < 0.001, η_p_^2^ = 0.68], a significant main effect of task type [*F*(1,29) = 9.12, *p* = 0.005, η_p_^2^ = 0.24], a significant interaction between distraction and task difficulty [*F*(2,58) = 4.78, *p* = 0.012, η_p_^2^ = 0.14], and a significant interaction between task difficulty and task type [*F*(2,58) = 16.99, *p* < 0.001, η_p_^2^ = 0.37]. The interaction between distraction and task type [*F*(2,58) = 2.06, *p* = 0.162, η_p_^2^ = 0.07] and the interaction between the three factors [*F*(2,58) = 0.02, *p* > 0.250, η_p_^2^ = 0.001] were not significant. For the CR data, the results showed a significant main effect of task difficulty [*F*(2,58) = 271.98, *p* < 0.001, η_p_^2^ = 0.90], a significant main effect of task type [*F*(1,29) = 4.43, *p* = 0.044, η_p_^2^ = 0.13], a significant interaction between distraction and task difficulty [*F*(2,58) = 8.64, *p* = 0.001, η_p_^2^ = 0.23], a significant interaction between distraction and task type [*F*(2,58) = 4.76, *p* = 0.037, η_p_^2^ = 0.14], and a significant interaction between task difficulty and task type [*F*(2,58) = 9.88, *p* = 0.001, η_p_^2^ = 0.25]. The main effect of distraction [*F*(2,58) = 0.01, *p* = 0.953, η_p_^2^ < 0.01] and the interaction between the three factors [*F*(2,58) = 0.58, *p* = 0.543, η_p_^2^ = 0.02] were not significant.

### Relation Between the RT and CR Data

Previous studies have found two observations in terms of the relations between the RT and CR data. First, for investigation of interference, the RTs have been suggested to be more sensitive than the CRs in revealing the interference effect (Beck and Lavie, 2005; Bahrami et al., 2007). Second, for the IC shape discrimination paradigm, it has been shown that the RT data paralleled the CR data in revealing the perceptual IC processing (Rubin et al., 1997; Kellman et al., 1998; Maertens, 2008). These relations between the RT data and CR data were also observed in the present study. First, the RT costs were overall more prominent than the CR costs in both the IC task (see Figure 3B) and the control RC task (see Figure 4B). Particularly, in the control RC task, a CR cost was lacking in all the three difficulty levels, while a strong RT cost was shown in the easy condition. Therefore, the present results were generally consistent with the previously found sensitivity of RTs in examining the interference effect relative to CRs. Meanwhile, previous studies used figures with RCs (Beck and Lavie, 2005; Bahrami et al., 2007), whereas the present study investigated figures with ICs and indicated that the CR data would be sensitive for revealing distraction of IC figures. Second, in both the IC and the control RC tasks, the CRs decreased, whereas the RTs increased with task difficulty, exhibiting the typical trade-off pattern between accuracy and speed. The CR costs and RT costs also changed in reversal directions as task difficulty increased. Therefore, in line with the previous finding, the present RT and CR data showed consistent patterns indicating modulation of task difficulty on perceptual processing. It is also worth noting that, given the overall consistency between the RT and CR data, detailed differences in terms of a statistical criterion did exist. For example, the interaction between distraction and task difficulty in the above two-way ANOVA in the IC task reached significance for the CR data but not for the RT data. The CR data would be considered to generally parallel the RT data (Rubin et al., 1997; Kellman et al., 1998; Maertens, 2008), given the overall pattern (clearly shown in Figures 3B, 4B) that the CR cost and the RT cost changed in reversed directions with task difficulty as discussed above.

## Discussion

The present study aimed to investigate interference of discrimination of Kanizsa-type ICs by a distractor. The results showed a significant RT cost in the easy and medium conditions and a significant CR cost in the easy condition, and the magnitude of the CR cost significantly reduced as task difficulty increased.

Attentional involvement in IC perception is an important topic in IC research, and previous research has focused on attraction of attention or modulation of attention allocation by ICs (Davis and Driver, 1994; Vuilleumier et al., 2001; Senkowski et al., 2005; Conci et al., 2007; Keane et al., 2011). The present study addressed the question of attentional engagement of IC processing by examining whether discrimination of ICs would be vulnerable to a distractor (Lavie, 1995, 2005; Forster and Lavie, 2008; Lavie and Dalton, 2014). The observed interference effect supports that attention is required for perception of ICs (Coren, 1972; Gregory, 1972; Rock and Anson, 1979; Wu et al., 2015). The interference effect was more likely to be found in the easier conditions, which was in agreement with the perceptual load theory that distractors’ interference is typically observed in the lower load condition (Lavie, 1995). In addition, a significant RT cost was observed for RCs in the easy condition, indicating some similarity between the distracting influences of ICs and RCs. The CR cost was not observed for RCs, which may partly be because the CR is less sensitive than the RT in revealing the interference effect (Beck and Lavie, 2005; Bahrami et al., 2007). The relation between the interference effects of ICs and RCs remains to be further clarified.

A difficulty in Kanizsa figure research is whether observers are processing the completed object or processing the rotation information of the inducers. To address this issue, the inducers of Kanizsa figures are often rotated; thus, the IC disappears. This control condition, although commonly adopted, still fails to provide a clear solution for the issue, since inducers are rotated in different manners for Kanizsa and control figures and subjects may perform Kanizsa tasks by simply relying on rotation information of the inducers (for review, see Seghier and Vuilleumier, 2006; Anderson, 2007). In one of our previous works (Yang et al., 2015), we further devised a new paradigm using a circle shape instead of the commonly used square or triangle shape. The contour fragments of a Kanizsa square or triangle figures provide clear cues for shape discrimination, whereas such discrimination cues were substantially diminished for circle shapes. The discrimination task was hard to perform by relying on rotation information of inducers of circle shapes, proving that the subjects were actually processing a completed object (Yang et al., 2015). The present study adopted the paradigm using circle shapes.

It is worth mentioning that the distractor in the current distraction design, i.e., the central dynamic patch, may not be entirely irrelevant to the peripheral IC discrimination task. For Kanizsa-type ICs, a surface surrounded by the contour is also perceived (Stanley and Rubin, 2003). Specifically, to the present task of discrimination between a thin circle contour and a short circle contour, a thin circle surface or a short circle surface would also be perceived. Therefore, when the dynamic patch was spatially separated from the contour, it was on the perceived surface. The present study was not designed to investigate the relation between contour processing and surface processing. We emphasize that the current findings and discussions are limited in terms of perception of Kanizsa-type IC figures in general and not with respect specifically to contours or surfaces. In the present design, efforts have been made so that the distractor was less relevant to the IC discrimination task, and future research would test the effect of a distractor that is salient enough and is entirely irrelevant to the task.

The present study investigated difficulties of the perceptual discrimination task as an experimental factor. Individual performance differences in each difficulty level were observed, consistent with previous studies examining IC perception (Ringach and Shapley, 1996; Pillow and Rubin, 2002) and perceptual load (Lavie, 1995). Individual difference is an important question in IC and attention research, such as the individual characteristics revealed in an IC learning task (Rubin et al., 1997). Individual performance differences in IC perception would be further addressed. For example, performances of subjects could be adjusted to specific criteria, which can also help dissociate variation in subjective performance from objective performance (Fleming et al., 2010).

Kanizsa figures represent a classic type of stimuli used in investigation of the role of attention in visual object processing. In Kanizsa figures, ICs can occur even when the supported physical boundaries are widely separated in space, and such long-range visual completion has great ecological relevance given the frequent occurrence of occlusion in natural scenes (Pillow and Rubin, 2002). Using Kanizsa-type ICs, a large variety of shapes can be formed and have been used in the literature (Seghier and Vuilleumier, 2006). Notably, it is surprisingly easy for the visual system to integrate separate physical contour fragments and perceive the various shapes formed by Kanizsa-type ICs (Gregory, 1972). In this regard, whether Kanizsa-type IC perception is a pre-attentive or automatic process could provide important insight into the understanding of attentional involvement in visual object processing (corresponding attention research of Kanizsa-type ICs has been introduced in more detail in section “Introduction”). Attention refers to the ability to focus on what is task-relevant while ignoring what is task-irrelevant, and a crucial question is how and particularly when this selective process takes place. Proposed as a solution to the long-standing early versus later selection debate, perceptual load theory suggests that perceptual load is a major determinant for visual attention (see Lavie and Tsal, 1994; Lavie, 1995 for thorough theoretical and methodological discussions). With respect to the current work, perceptual load theory indicates that the attentional requirement of Kanizsa-type IC perception may depend on perceptual load, which has not been addressed in previous Kanizsa-type IC research. More specifically, to further clarify the role of attention in Kanizsa-type perception, perceptual load theory suggests examining the extent to which perceiving Kanizsa-type ICs in the face of irrelevant distractors depends on the perceptual load of attended Kanizsa-type ICs. The present study was inspired by perceptual load theory and, accordingly, designed a distraction paradigm with varied perceptual loads.

In summary, the present study used a distraction paradigm to investigate involvement of attention in perception of Kanizsa-type ICs. The results showed that the interference effect was more likely to be observed in the easy conditions. The finding suggests that high-level cognitive processes are engaged in perception of ICs and that the attentional involvement in IC processing is associated with perceptual load.

## Data Availability Statement

The datasets generated for this study are available on request to the corresponding author.

## Ethics Statement

The studies involving human participants were reviewed and approved by the Institutional Review Board of the Psychology Department at Sun Yat-sen University. The patients/participants provided their written informed consent to participate in this study.

## Author Contributions

JY and XW designed the research and wrote the manuscript. JY and HW performed the research. JY analyzed the data. LS, QW, and XM were involved in the discussion of the data. All authors contributed to the article and approved the submitted version.

## Conflict of Interest

The authors declare that the research was conducted in the absence of any commercial or financial relationships that could be construed as a potential conflict of interest.

## Footnotes

1 http://psychtoolbox.org

2 http://www.microsoft.com
