# Supplementary material for: Interference of Illusory Contour Perception by a Distractor
Source: Front Psychol. 2021 Jun 11;12:526972. doi: 10.3389/fpsyg.2021.526972 (PMC8231925; doi:10.3389/fpsyg.2021.526972)
Supplement: Supplementary file 1 [file Data_Sheet_1.PDF]

Title: Interference of illusory contour perception by a distractor

Authors: Junkai Yang [1,3], Lisen Sui [2], Hongyuan Wu [3], Qian Wu [3], Xiaolin Mei [3], Xiang Wu [3*]

Supplementary materials

Fig. S1-S3; Table S1-S2; Supplementary Text.

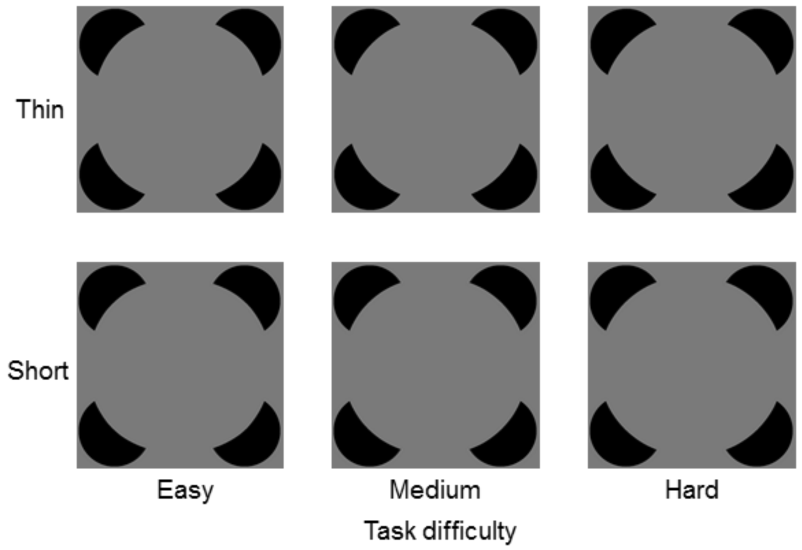

Fig. S1. Illustration of illusory contour stimuli in all levels of task difficulty. Conventions are as in Fig. 1.

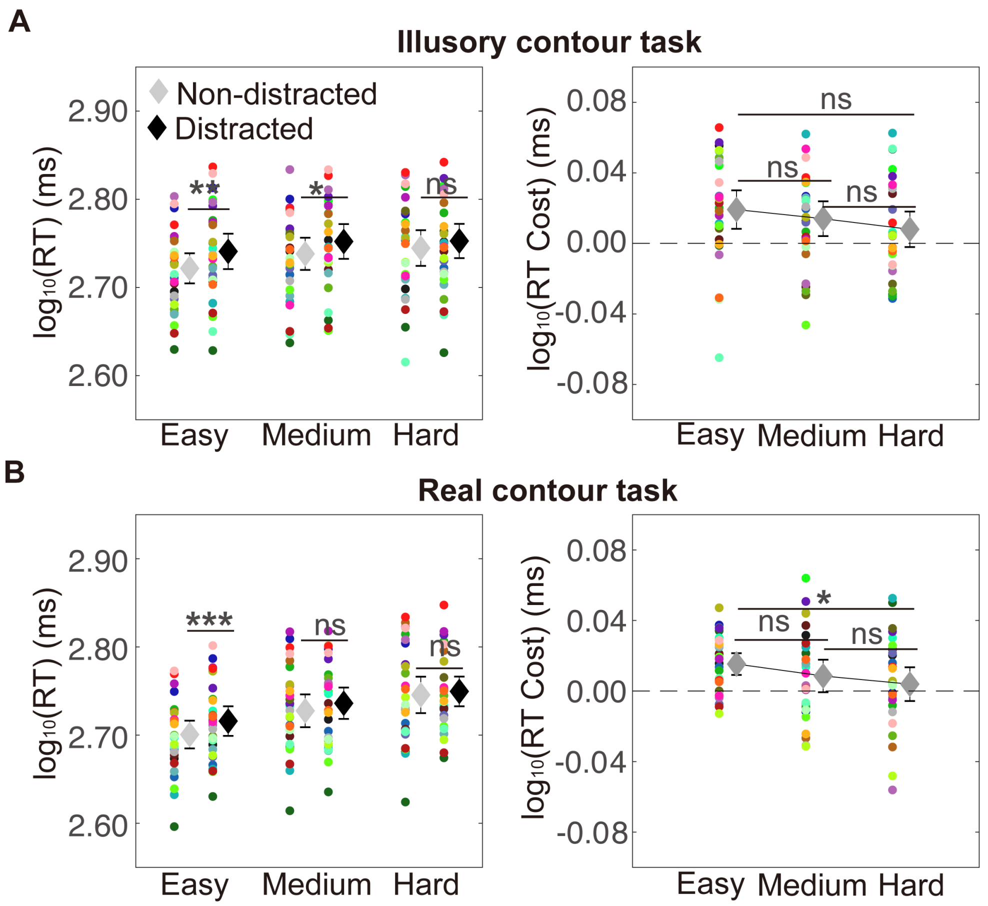

Fig. S2. Log Reaction times in the main and exploratory experiments. The log(RT) results were consistent with the results shown the main text. Conventions are as in Fig. 3 and Fig. 4.

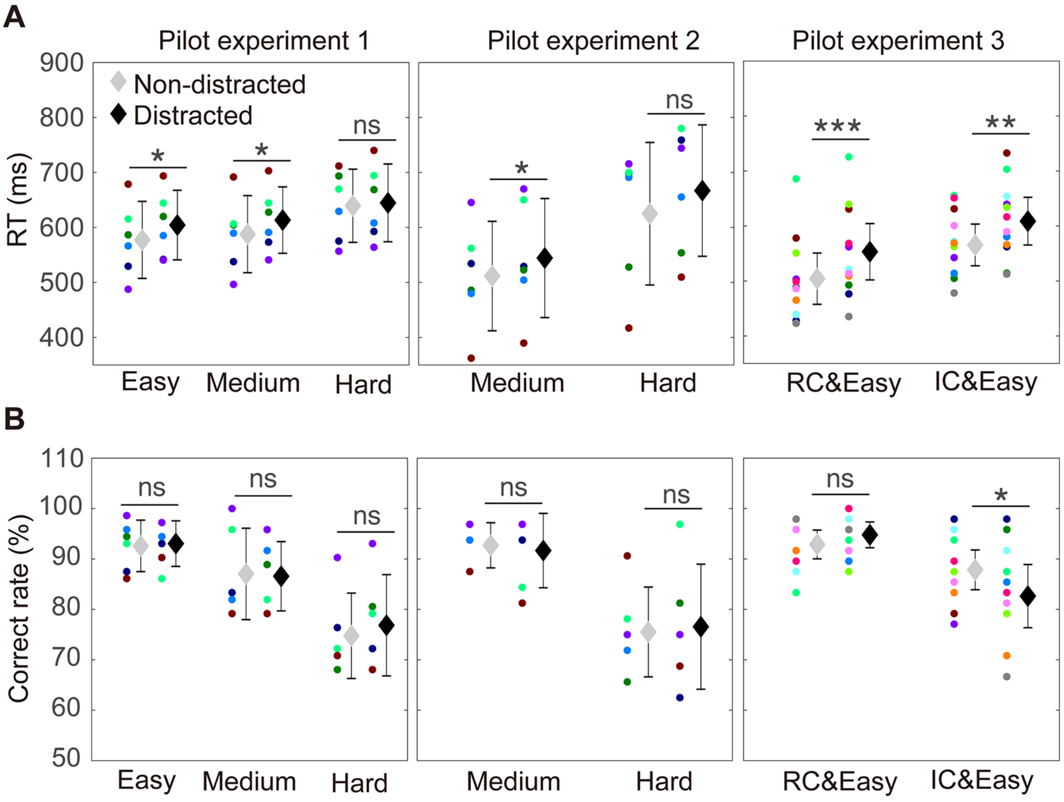

Fig. S3. Illustration of results in Pilot experiments 1 to 3. Conventions are as Fig. 3.

Task	Difficulty	*Mean difference*	*t*	*P corrected*	95% CI
		**Pilot experiment 1**			
RC	Easy	26.94	4.36	.021	[11.05, 42.82]
	Medium	25.61	3.74	.039	[8.01,43.22]
	Hard	5.23	.56	> .250	[-18.95, 29.42]
		**Pilot experiment 2**			
RC	Medium	32.75	2.63	.047	[.68, 64.82]
	Hard	41.97	2.21	.078	[-6.82, 90.77]
		**Pilot experiment 3**			
RC	Easy	49.00	6.58	<.001	[32.62, 65.39]
IC	Easy	43.27	3.38	.006	[15.06, 71.48]

Table S1. Comparisons between RTs of distracted and non-distracted responses in Pilot experiments 1 to 3. Other conventions are as in Table 1.

Task	Difficulty	*Mean difference*	*t*	*P corrected*	95% CI
		**Pilot experiment 1**			
RC	Easy	-.46	-.25	> .250	[-5.31, 4.39]
	Medium	.46	.14	> .250	[-8.36, 9.29]
	Hard	-2.08	-.77	> .250	[-9.03, 4.86]
		**Pilot experiment 2**			
RC	Medium	1.04	.36	> .250	[-6.34, 8.42]
	Hard	-1.04	-.17	> .250	[-17.06, 14.98]
		**Pilot experiment 3**			
RC	Easy	-.02	-1.14	> .250	[-.06, .02]
IC	Easy	.05	2.49	.030	[.01, .10]

Table S2. Comparisons between CRs of distracted and non-distracted responses in Pilot experiments 1 to 3. Other conventions are as in Table 1.

Supplementary Text

Pilot experiments

Pilot experiment 1

Pilot experiment 1 was similar to the formal RC task in the main text with the following differences: (1) each stimulus type was measured 96 times; (2) rotation angles of 4.8°, 3.6°, and 2.4° were used for the easy, medium, and hard conditions, respectively.

Six participants (two males, mean age ± *SD* 24.5 ± 3.7 years) participated in this experiment. The results are shown in Fig. S3 (the first column). For the RTs, a two-way ANOVA with within-subject factors of distraction (without or with the distractor) and task difficulty (easy, medium and hard) were conducted, which showed a significant main effect of distraction ($F(1, 5) = 13.29$, $p = .015$, $\eta_p^2 = .73$), indicating the interference effect; and a significant main effect of task difficulty ($F(1, 5) = 51.25$, $p < .001$, $\eta_p^2 = .91$), indicating the effect of modulation of task difficulty. The interaction between the two factors was not significant. (Comparisons between RTs of distracted and non-distracted responses are listed in Table S1). For the CRs, a two-way ANOVA with within-subject factors of distraction (without or with the distractor) and task difficulty (easy, medium and hard) showed a significant main effect of task difficulty ($F(1, 5) = 33.25$, $p = .001$, $\eta_p^2 = .87$). The main effect of distraction and the interaction between the two factors were not significant (Comparisons between CRs of distracted and non-distracted responses are listed in

Table S2).

These results indicated the validity and effectiveness of the interference of contour discrimination by a centrally presented dynamic patch and the modulation of task difficulty in the current design.

Pilot experiment 2

Pilot experiment 2 was similar to Pilot experiment 1, and the difference was that an epoch/block design (i.e., different types of stimuli were not randomly mixed, but were arranged in separate epochs in a block) was adopted in Pilot experiment 2 (Pillow & Rubin, 2002). Two levels of task difficulty (medium and hard levels with rotation angles of 3.6° and 2.4°, respectively) were tested in two separated blocks. Each block was composed of alternation between epochs of non-distracted trials and epochs of distracted trials. There were four alternations in each block and each epoch contained eight trials.

Six participants (one male, mean age ± *SD* 23.3 ± 3.6 years) participated in this experiment. The results are shown in Fig. S3 (the second column). For the RTs, a two-way ANOVA with within-subject factors of distraction (without or with the distractor) and task difficulty (medium and hard) showed a significant main effect of distraction ($F(1, 5) = 8.77$, $p = .031$, $\eta_p^2 = .64$), indicating the interference effect; and a significant main effect of task difficulty ($F(1, 5) = 20.76$, $p = .006$, $\eta_p^2 = .81$),

indicated the effect of manipulation of task difficulty. The interaction between the two factors was not significant. (Comparisons between RTs of distracted and non-distracted responses are listed in Table S1). For the CRs, a two-way ANOVA with within-subject factors of distraction (without or with the distractor) and task difficulty (easy, medium and hard) showed a significant main effect of task difficulty ($F(1, 5) = 17.28$, $p = .009$, $\eta_p^2 = .78$). The main effect of distraction and the interaction between the two factors were not significant (Comparisons between CRs of distracted and non-distracted responses are listed in Table S2).

These results were consistent with the results in Pilot experiment 1, (1) further supporting the validity and effectiveness of the interference and the task-difficulty modulation in the current design, and (2) suggesting that the interference and the task-difficulty modulation in the current design would be observed in either a randomly mixed design or an epoch/block design.

Pilot experiment 3

Pilot experiment 3 was similar to Pilot experiment 2 except that (1) both real contours and illusory contours were investigated, and (2) only the easy condition with a rotation angle of 4.8° was used. Real and illusory contours were arranged in two separated sessions.

Twelve participants (one male, mean age ± *SD* 23.3 ± 1.5 years) participated in this experiment. The results are shown in Fig. S3 (the third column). For the RTs, a

two-way ANOVA with within-subject factors distraction (non-distracted and distracted) and factor contour type (RC and IC) were performed. The results showed a significant main effect of distraction ($F(1, 11) = 28.75$, $p < .001$, $\eta_p^2 = .73$), indicating the interference effect; a significant main effect of contour type ($F(1, 11) = 18.86$, $p = .001$, $\eta_p^2 = .63$), indicating that participants responded slower to ICs than to RCs, which was consistent with previous reports that shape discrimination performance was poorer for ICs than for RCs under the same level of shape deformation (Murray, Imber, Javitt, & Foxe, 2006; Ringach & Shapley, 1996). The interaction between the two factors was not significant. (Comparisons between RTs of distracted and non-distracted responses are listed in Table S1). For the CRs, a two-way ANOVA with within-subject factor distraction (non-distracted and distracted) and factor contour type (RC and IC) showed a significant main effect of contour type ($F(1, 11) = 12.69$, $p = .004$, $\eta_p^2 =.54$) and a significant interaction between the two factors ($F(2, 22) = 8.33$, $p = .015$, $\eta_p^2 = .43$).

These results were consistent with the results in Pilot experiments 1 and 2, (1) further supporting the validity and effectiveness of the interference and the task-difficulty modulation in the current design, and (2) suggesting that the interference and the task-difficulty modulation in the current design would be suitable for investigation of both real and illusory contours. In addition, the interference effect of the IC task in Pilot experiment 3 helped conduct priori power analysis for the formal experiments.

Taken together, while the pilot experiments had limitations, such as the small sample size in Pilot experiments 1 and 2 and only easy condition was examined in Pilot experiment 3, they provided helpful information for conducting later formal experiments, as described above.

References

Murray, M. M., & Herrmann, C. S. (2013). Illusory contours: a window onto the neurophysiology of constructing perception. *Trends in Cognitive Sciences*, *17*(9), 471–481.

Wu, X., He, S., Bushara, K., Zeng, F., Liu, Y., & Zhang, D. (2012). Dissociable neural correlates of contour completion and contour representation in illusory contour perception. Human Brain Mapping, 33(10), 2407–2414.
